# Genome Sequences of *Brucella abortus* and *Brucella suis* Strains Isolated from Bovine in Zimbabwe

**DOI:** 10.1128/genomeA.01063-14

**Published:** 2014-10-23

**Authors:** Betty Ledwaba, Joseph Mafofo, Henriette van Heerden

**Affiliations:** a University of Pretoria, Faculty of Veterinary Science, Department of Veterinary Tropical Diseases, Onderstepoort, South Africa; b Agricultural Research Council-Biotechnology Platform, Onderstepoort, South Africa; c Genomics Research Institute (GRI), University of Pretoria, Pretoria, South Africa

## Abstract

This is a report of whole-genome sequences of a *Brucella abortus* strain and two *Brucella suis* strains isolated from bovine in Zimbabwe. These strains were selected based on their origin and data obtained when using multiplex PCR assays, then sequenced using next-generation sequencing technologies.

## GENOME ANNOUNCEMENT

*Brucella* species are small Gram-negative coccobacilli that cause the zoonotic disease brucellosis. The disease has a negative impact on global economy and public health as it causes substantial losses of livestock and affects the livelihood of communities relying on their livestock for survival. Currently eight terrestrial species, *Brucella abortus, B. melitensis, B. suis, B. canis, B. ovis, B. neotomae, B. inopinata*, and *B. microti*, as well as two marine strains, *B. ceti* and *B. pinnipedialis*, are recognized (1–3). Species are classified on the basis of phenotypic and genotypic properties, host preference, and pathogenicity (4). In Zimbabwe, only *B. abortus* and *B. melitensis* have been reported to cause brucellosis in animals (5).

In this report, we present the whole-genome sequences of three field strains, ZW043 (batch number 43 at Central Veterinary Laboratory [CVL], Zimbabwe), ZW046 (batch number 46 at CVL), and ZW053 (batch number 53 at CVL), that were isolated from bovine in Zimbabwe. Isolates were selected on the basis of the data obtained with multiplex PCR assays (Bruce-ladder [6], Suis-ladder [7], and AMOS [8, 9]), which identified ZW043 and ZW046 as *B. suis* and ZW053 as *B. abortus* performed prior to sequencing. The genomic DNA was sequenced using Illumina Miseq (Illumina) paired-end sequencing technology. A Nextera DNA sample preparation kit (Illumina) was used for the sequencing library preparation and DNA fragments in the 500- to 1,000-bp range were selected. The quality of the data was checked with FastQC. After trimming and merging the overlapping sequence reads, *de novo* sequence assembly was performed using Abyss-pe version 1.3.6 (10) with k-mer length set at 64. The genome assemblies of ZW043, ZW053, and ZW046 were in the order 3.4, 3.3, and 3.4 Mb, respectively. Each genome assembly was ordered into pseudomolecules (chromosomes) using ABACAS (11). The ordered chromosomes were annotated using RAST (12). Totals of 3,370, 3,414, and 3,383 coding sequences (CDSs) as well as 63, 52, and 51 tRNAs were, respectively, predicted from ZW043, ZW046, and ZW053. CLC Genomic Workbench version 5.5 (CLC Bio) was used to map the reads to references as well as performing the variant detection and comparisons. Comparative genome analyses showed that ZW043 and ZW046 were most similar to *B. suis* bv. 1 strain 1330 (13) whereas ZW053 was most similar to *B. abortus* bv. 1 strain 9-941 (14) and all genomes had an average GC content of 57%. This report confirms the presence of *B. abortus* and *B. suis* in bovine. *Brucella suis* mainly causes brucellosis in pigs as a primary host but has been reported in other host including bovine (4). This is the first report of *B. suis* in Zimbabwe. Comparing these sequences further with other *Brucella* genomes will present insight into the behavior of this wide range genus.

### Nucleotide sequence accession numbers.

Genome sequences of chromosome 1 and 2 for ZW043, ZW046, and ZW053 are available in GenBank under the following respective accession no.: CP009094; CP009095; CP009096; CP009097; CP009098; CP009099.
